# Family-based association analysis of alcohol dependence implicates KIAA0040 on Chromosome 1q in multiplex alcohol dependence families

**DOI:** 10.4236/ojgen.2013.34027

**Published:** 2013-12

**Authors:** Shirley Y. Hill, Bobby L. Jones, Nicholas Zezza, Scott Stiffler

**Affiliations:** Department of Psychiatry, University of Pittsburgh School of Medicine, Pittsburgh, USA

**Keywords:** KIAA0040, Alcohol Dependence, Multiplex, Families

## Abstract

**Background:**

A previous genome-wide linkage study of alcohol dependence in multiplex families found a suggestive linkage result for a region on Chromosome 1 near microsatellite markers D1S196 and D1S2878. The KIAA0040 gene has been mapped to this region (1q24 - q25). A recent genome-wide association study using SAGE (the Study of Addiction: Genetics and Environment) and COGA (Collaborative Study on the Genetics of Alcoholism) found five SNPs within the KIAA0040 gene significantly associated with alcohol dependence. A meta-analysis using data from these sources also found the KIAA0040 gene significantly associated with alcohol dependence.

**Methods:**

Using family data consisting of 1000 individuals with phenotypic data (762 with both phenotype and DNA), finer mapping of a 0.3 cM region that included the KIAA0040 gene and a flanking gene was undertaken using SNPs with minor allele frequency (MAF) ≥ 0.15 and pair-wise linkage disequilibrium (LD) of r^2^ < 0.8 using the HapMap CEU population.

**Results:**

Significant FBAT p-values were observed for six SNPs, four within the KIAA0040 gene (rs2269650, rs2861158, rs1008459, rs2272785) and two adjacent to KIAA0040 (rs10912899 and rs3753555). Five haplotype blocks of varying size were identified using HAPLOVIEW. Analysis using the haplotype-based test function of FBAT revealed one two-SNP block (rs1008459-rs2272785) associated with alcohol dependence. This block showed a pattern of transmission in which one haplotype, CT, with a frequency of 0.577 was found to be over-transmitted to affected offspring (p = 0.017) while another haplotype, AG, with a frequency of 0.238 was found to be under-transmitted to affected offspring (p = 0.006). A three-SNP block (rs1008459-rs2272785-rs375355) showed an overall significant association (p = 0.011) with alcohol dependence with the haplotype ACT over-transmitted to affected offspring (p = 0.016) and the haplotype GAG under-transmitted (p = 0.002).

**Conclusions:**

Family-based association analysis shows the KIAA0040 gene significantly associated with alcohol dependence. The potential importance of the KIAA0040 gene for AD risk is currently unknown. However, the present results support earlier findings from a genome-wide association study.

## 1. INTRODUCTION

Alcohol dependence is a complex disorder that is characterized by psychological and physical dependence and is often accompanied by chronic consumption of hazardous levels of ethanol. Excessive use of alcohol is the third leading cause of preventable death [1] in the US. The economic and social costs have been estimated to be $184 billion due to alcohol-related accidents, lost productivity, incarceration and other alcohol related morbidity [2]. In spite of the fact that the use of alcohol is quite common, a smaller proportion of the population drink in sufficient quantity and with associated health, family and work-related problems to be considered alcohol dependent AD. The one-year prevalence of AD in the US is 3.8% [3]. The lifetime prevalence of AD has been estimated at 12.5% [4]. Prevalence among male respondents ages 15 – 54 has been reported to be higher with 20.1% of men and 8.2% of women meeting criteria for alcohol dependence [5]. There is now evidence that those individuals with the greatest propensity for AD may carry an increased genetic risk for developing alcohol dependence.

Although there is considerable heritability for alcohol dependence (0.49 – 0.64) in males [6,7] and females (0.56 – 0.59), [8,9] few genes have been identified that reliably confer susceptibility. However, studies employing well-designed sampling strategies over sample families with a high density of cases have revealed important clues for gene finding as seen in the Collaborative Study on the Genetics of Alcoholism (COGA) studies [10,11]. Genome-wide association (GWAS) studies have also revealed potentially important loci but require large samples to detect loci having genome-wide significance. A meta-analysis of two GWAS studies of alcohol dependence totaling 4979 cases and controls has identified three loci with statistical significance of p < 5 × 10^−7^ [12].

In a genome-wide scan of multiplex families ascertained through a pair of affected probands [13], we found evidence for linkage in multiple chromosomal regions. The present report is based on efforts to follow up on linkage findings for a region on Chromosome 1q23.3 to 1q25.1 that includs a maximal LOD score of 3.46 (p = 0.002) at marker D1S196 and at an adjacent marker D1S2878 with a LOD value of 3.45 (p = 0.002). A previous follow-up of this region revealed significant family- based association for the astrotactin neuronal protein (ASTN1) gene [14]. Two nearby genes, KIAA0040 and TNN have been mapped to 1q25.1 and have been recently identified in genome-wide association analyses as being significantly related to alcohol dependence [12,15]. Accordingly, a study of the TNN/KIAA0040 region was undertaken.

## 2. MATERIALS AND METHODS

### 2.1. Study Sample

Written consent was obtained from all members of the multiplex families who participated in the study after the nature and purpose of the study was fully explained to them. The consent forms used in the study were approved by the University of Pittsburgh Institutional Review Board.

### 2.2. Multiplex Families

Multiplex families were selected on the basis of the presence of a pair of alcohol dependent brothers or sisters. The probands were selected from among individuals in treatment for alcohol dependence in the Pittsburgh area. Probands were eligible if they met DSM-III criteria for AD and had a same sex sibling who similarly met criteria for AD. Families were excluded if the probands or any first-degree relative were considered to have a primary diagnosis of drug dependence (preceded alcohol dependence onset by at least 1 year), or the proband or first-degree relative met criteria for schizophrenia, or a recurrent major depressive disorder. Probands and relatives with mental retardation or physical illness precluding participation were excluded. Complete details regarding participant selection may be seen in Hill *et al*. [13]. The majority of probands (80%) had three or more siblings who contributed DNA, consented to a clinical interview, and provided family history. These large sibships resulted in a total of 648 sib pairs within the proband generation. Across the generations, an average of 5.7 individuals per family was genotyped.

### 2.3. Generation I and II Diagnoses

All proband pairs and their cooperative relatives (siblings and parents) were personally interviewed using a structured psychiatric interview (Diagnostic Interview Schedule [DIS]). The DIS provides good reliability and validity [16] for alcohol dependence and alcohol abuse by DSM-III and IIIR criteria [17,18] the diagnostic criteria in place when the study began. The DIS also provides an alcoholism diagnosis by Feighner Criteria [19].

### 2.4. Generation III—Young Adult Assessment for DSM-IV Diagnoses

With the initiation of a third generation follow-up, offspring who had reached their 19th birthday were assessed using the Composite International Diagnostic Interview (CIDI) [20] to determine the presence or absence of a DSM-IV Axis I diagnosis. The CIDI-SAM (Substance Abuse Module) [21] was also administered in order to determine quantity, frequency, and pattern of drug and alcohol use. Interrater reliability for interviewers on the diagnostic instruments used in this study exceeded 90%.

### 2.5. SNP Selection

Previously, we carried out a genome-wide linkage analysis finding potentially important linkage results for multiple regions including Chromosome 1 [13]. Our study included genotyping in a 26.6 cM region on Chromosome 1 that centered on the microsatellite marker D1S196. A LOD score of 3.46 was obtained using a binary alcohol dependence phenotype and including relevant covariates (age, gender and the personality variable Constraint). Constraint from the Multidimensional Personality Questionnaire measures tendencies to inhibit impulse expression, rejection of unconventional behavior, and risk taking and with genetic variance of 0.58 in twins reared apart [22].

In order to investigate the region further, SNPs were chosen with minor allele frequency (MAF) ≥ 0.15 and pair-wise linkage disequilibrium (LD) of r^2^ < 0.8 using the HapMap CEU population at approximately 1 cM intervals in this region. The genotyping and analysis was completed in three stages. First, we focused on a 19 cM region extending from rs7522166 to rs2816187. This region, bounded by these SNPs was chosen because rs7522166 is 7 cM proximal to D1S196 and rs2816187 is 13 cM distal to D1S196. We genotyped 18 SNPs at approximately 1 cM intervals in this region. Analysis of these 18 SNPs revealed the greatest statistical significance for rs228008 located in the ASTN1 gene. Finer mapping of this gene at an average distance of 28.9 kb using twelve additional SNPs confirmed the significant result obtained for rs228008 [14]. Because two nearby genes, KIAA0040 and TNN (1q25.1) have shown highly significant association with alcohol dependence in a GWAS study [15], a study of this region was planned. A total of 18 SNPs were selected for genotyping with 9 SNPs selected to cover a 0.3 cM region extending from rs12094153 to rs3753555 covering the TNN/KIAA0040 region at intervals of no greater than 5 kb, with 8 SNPS selected for their presence within the KIAA0040 gene. The SNPs selected for presence within the gene were chosen based on the reports of Wang *et al*. [12] and Zuo *et al.* [15]. Specifically, from among the SNPs evaluated by Zuo *et al*. [15], the five SNPs having the best statistical significance were chosen (rs6701037, rs6425323, rs1057302, rs1057239, and rs1894709). These SNPs were also reported to be significantly related to alcohol dependence in the Wang *et al*. [12] meta-analysis.

### 2.6. DNA Isolation and Genotyping

Genomic DNA was extracted from whole blood with a second aliquot prepared for EBV transformation and cryopreservation. PCR conditions were as described in Hill *et al*. [13]. Genotyping was completed on a Biotage PSQ 96 MA Pyrosequencer (Biotage AB, Uppsala, Sweden). Each polymorphism was analyzed by PCR amplification incorporating a biotinylated primer. Thermal cycling included 45 cycles at an annealing temperature of 60°C. The Biotage workstation was used to isolate the biotinylated single strand from the double strand PCR products. The isolated product was then sequenced using the complementary sequencing primer.

### 2.7. Quality Control

SNP genotyping quality control involved ongoing monitoring of SNP signals provided by Qiagen software. Output is provided using three categories for each SNP: pass, fail and check. Data analysis was performed for only those signals meeting the “pass” criterion. Signals that failed or were returned as needing further checking were rerun. If after 3 attempts the SNP did not meet the “pass” criterion, it was eliminated from the analysis and another SNP chosen as a replacement.

### 2.8. Statistical Methods

The sample included 133 pedigrees consisting of 1000 individuals (49% male and 51% female). Among the 1000 subjects, 542 were affected, 436 were unaffected, and 22 had unknown status.

### 2.9. Mendelian Inconsistency

The PedCheck program [23] was used to evaluate individual SNPs for Mendelian inconsistencies based on the pedigree structures. As a result of the evaluation, 36 marker genotypes from among the 13,656 were coded as missing to resolve the reported inconsistencies.

### 2.10. Hardy-Weinberg Equilibrium (HWE)

Estimates of population allele frequencies were calculated using MENDEL version 11 [24]. Files required by the MENDEL program were generated via the program Mega2 [25]. Marker allele frequencies were tested for departures from Hardy-Weinberg equilibrium using the allele frequency option in MENDEL. None of the 18 SNPs analyzed were found to have p-values below the Bonferroni adjusted threshold (<0.003) that would indicate significant HWE departures.

### 2.11. Genetic Maps

The Genetic Map Interpolator (GMI) software [26] was used to retrieve current physical map positions from Ensembl (Ensembl 68). These physical positions were then used to linearly interpolate genetic map positions based on the Rutgers Combined Linkage-Physical Map [27,28].

### 2.12. Family-Based Association Test (FBAT)

Transmission rates of marker alleles were examined using the family-based association test program, FBAT [29,30], assuming an additive genetic model with robust variance estimation (−e option) to account for the relatedness. This family-based method is a generalization of the transmission disequilibrium test (TDT) [31], which provides a valid test of association even if admixture is present. FBAT converts each pedigree into nuclear families, which are then treated as independent families for the test statistic calculation. Informative families consisting of parent-child trios are utilized in the FBAT analysis.

Generation I and II individuals were coded as affected if they met criteria for alcohol dependence by DSM-III criteria. Generation III individuals were coded as affected if they met criteria for alcohol abuse or dependence, or drug abuse or dependence. Choice of this broader phenotype for the third generation was based on the greater prevalence of drug use disorders in the third generation. Also, Generation I and II individuals had been selected for the family study based on the presence of primary alcohol dependence (if drug dependence was presence it must have followed the alcohol dependence diagnosis by one year). However, analyses were also conducted using an alcohol abuse/dependence phenotype for the third generation.

### 2.13. Gamete-Competition (GC)

We also considered the gamete-competition model [32], a generalization of the transmission disequilibrium test (TDT), to investigate association of marker alleles with alcohol dependence. The gamete-competition model can be used to test for differences in transmission of marker alleles to affected individuals.

### 2.14. Haplotype Analysis

Linkage disequilibrium (LD) analysis was performed using the HAPLOVIEW program version 4.2 [33]. The LD block structure was defined by calculating D’ values pairwise between SNPs. SNP haplotype blocks were created using the HAPLOVIEW default block determination method [34]. Additionally, a sliding window approach was used to identify two and three SNP blocks in order to insure that any blocks within larger haplotype blocks could be analyzed. Haplotype blocks were investigated for family-based association with affected status. A within-family association analysis between alcohol dependence and the revealed haplotypes was performed using haplotype FBAT [35] assuming an additive genetic model and using a robust estimate of variance (−e option).

## 3. RESULTS

### 3.1. Association Results

**Figure 1:**
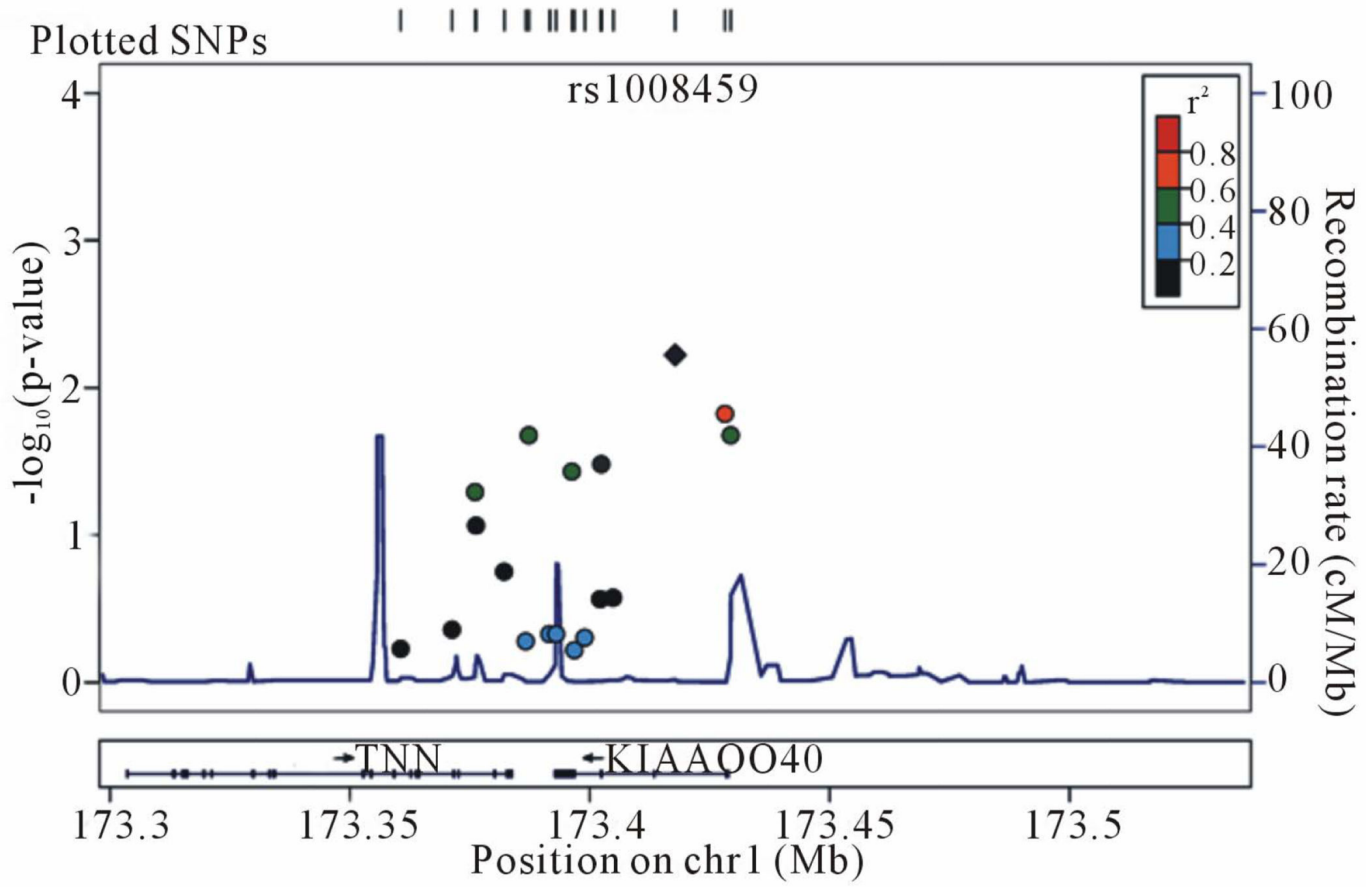
Association plot (−log_10_ of the p values from FBAT) for SNPs within 120 kb of rs1008459, the SNP with the maximum association observed.

Analysis of 18 SNPs covering a 68.8 Kb region on Chromosome 1 extending from rs12094153 to rs3753555 revealed six SNPs associated with alcohol dependence with significant FBAT p values (rs2269650, rs2861158, rs1008459, rs2272785, rs10912899, rs3753555). The SNP showing the most significant association with alcohol dependence affected status was rs1008459 (FBAT p = 0.006) located within intron 2 of KIAA0040. Four SNPs are within the KIAA0040 gene. Two of these were also found to be significant using Gamete Competition (GC) analyses. Results for the FBAT and GC analyses are summarized in Table 1. LocusZoom [36] was used to generate a plot of the association test results (Figure 1).

### 3.2. Haplotype Analysis

**Figure 2:**
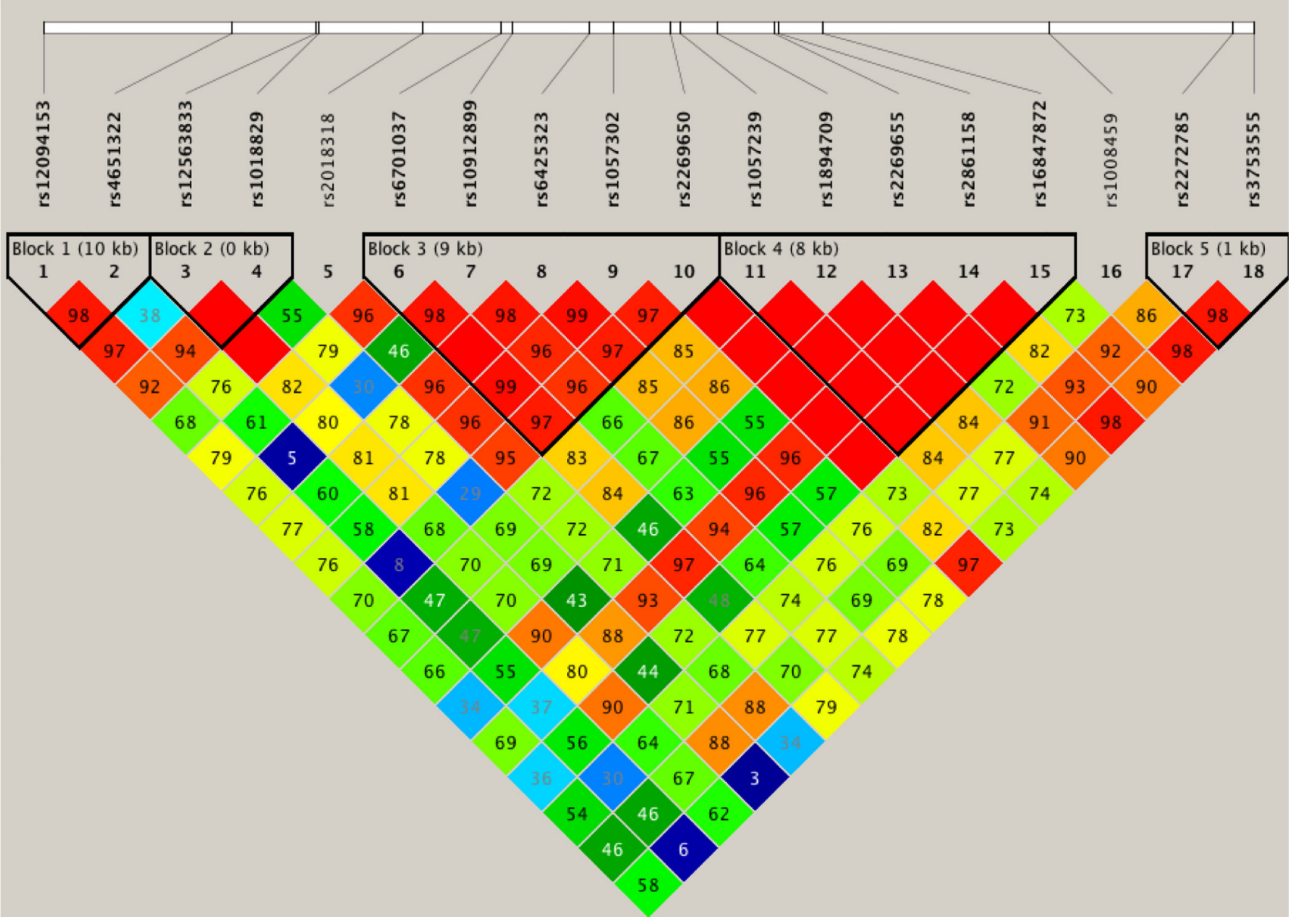
Linkage disequilibrium analysis was performed using HAPLOVIEW (version 4.2). The block structure was defined by calculating D’ values pairwise between SNPs. One two SNP block was identified containing rs2272785 and rs3753555 that showed statistical significance with alcohol dependence. The haplotype CT with a frequency of 0.577 was found to be over-transmitted to affected offspring (p = 0.017). Block AG with frequency of 0.238, was under-transmitted to affected offspring (p = 0.006).

Five haplotype blocks were identified by HAPLOVIEW. Pairwise linkage disequilibrium between the SNPs and the LD block structure are shown in Figure 2. Haplotype analyses were performed using two alternative phenotypes for Generation III (see Table 2). The gender distribution by generation may be seen in Table 3. Results of the haplotype analysis can be seen in Table 4. One two SNP block (Block 5) which consisted of one SNP within the KIAA0040 gene (rs2272785) and an adjacent SNP (rs3753555) showed an association with affected status with a p value of 0.041 using a broader SUD phenotype and 0.034 when restricted to alcohol abuse or dependence only. In Block 5, the haplotype CT with a frequency of 0.577, was found to be over-transmitted to affected offspring (p = 0.017) while the haplotype block AG with a frequency of 0.238, was found to be under-transmitted to affected offspring (p = 0.006). This analysis was first performed using affected status for Generation III to include any SUD (alcohol abuse or dependence, or drug abuse or dependence). Re-analysis using alcohol dependence only as the affected phenotype for Generation III resulted in minor alterations in significance (p = 0.044 and p = 0.009, respectively).

None of the other four blocks identified by HAPLOVIEW were found to be associated with alcohol dependence.

Based on results from our sliding window analysis, we find an association for alcohol dependence for a three-SNP block that includes the previously identified two-SNP block and includes rs1008459 and rs2272785 along with rs3753555. This three-SNP block showed a overall significant association (p = 0.012) with SUD with an individual p-value for GAG of 0.002. This result obtained with third generation offspring coded as affected, whether alcohol dependent or having SUD, was confirmed using third generation codes as affected when only alcohol dependence was present showed an overall probability of (p = 0.011) and a haplotype specific p-value for GAG of 0.003.

## 4. DISCUSSION

Within-family association (FBAT and GC) analyses were performed for 18 SNPs in a region of Chromosome 1. Based on the FBAT within-family association analyses, our results suggest that variation in the KIAA0040 gene is associated with risk for alcohol dependence in families with multiple cases of alcohol dependence. These results support findings from a genome-wide association study [15] and a meta-analysis that includs data from studies that utilizes both case/control and within family association analyses for alcohol dependence for SNPs within the KIAA0040 gene [12].

Zuo *et al*. [15] reported significant results for five SNPs within the KIAA0040 gene (rs10572239, rs1894709, rs6701037, rs6425323, rs1057302). It is noteworthy that this GWAS study also finds one SNP having Cis-Acting regulatory effects and the rs2269650 SNP with a p value of 8.6 × 10^−5^. This SNP showed significant results in the present family-based association analysis as well. However, the five top ranked SNPs reported in Zuo *et al*. [15] were not significant in our within-family association analysis. Two of these SNPs lie in the region proximal to the KIAA0040 gene (rs6701037 and rs6425323) and distal to the TNN gene while three other SNPs lie in Exon 5 (rs1057302 and rs1057239) and intron 4 (rs1894709). Consideration of the meta-analysis of family data provided by Wang *et al*. [12] shows a replication in the present family data for one SNP (rs1008459) with a reported FBAT value of 0.0367. However, it should be noted that three SNPs (rs6701037, rs2269655, and rs6425323) that reached genome-wide significance in the analysis of Wang *et al*. [12] were not significant in the present study. However, our results for rs10912899 which lies between two of these SNPs, rs6701037 and rs6425323, did show significance (p = 0.02) based on our FBAT analysis.

Haplotype analysis revealed one two-SNP block with a p-value of 0.006 and one three-SNP block with a p-value of 0.002. The Zuo *et al*. [15] SNPs showing genomewide significance appear to cluster in Exon 5, whereas the current results also suggest the importance of Exon 4 (rs2861158) and intron 2 where we found a two-SNP and a three-SNP haplotype respectively with p-values suggesting their importance.

The biological significance of the current findings is unknown because the KIAA0040 gene encodes a protein whose function is unknown. There is evidence that the KIAA0040 protein product may represent one of the tumor antigens expressed on colorectal cancer cells and recognized by tumor reactive T-cells (CT28 line) [37]. The KIAA0040 gene is flanked by two genes TNN and TNR with a plausible role in alcohol dependence. A previous study has reported significance of SNPs within the TNN gene and AD [15]. Because the TNN gene lies only 8.9 kb from KIAA0040, the TNN gene is of interest. Also, the TNN gene encodes a protein, tenascin-N, which is involved in neurite outgrowth and cell migration in the hippocampus [38]. Weaker evidence for a role of the TNN gene in AD was seen in the present analysis with two SNPs (rs12563833 and rs1018829) showing marginal FBAT significance.

The present results should be considered in the context of some limitations. Although this study represents a follow-up on a linkage peak originally reported for this region of Chromosome 1, the peak observed was relatively large [13]. Because the peak was broad, it may be expected that a number of genes are within this peak. KIAA0040 was not included in our original planned analysis as its function has not been defined. However, KIAA0040 has shown genome-wide significance for alcohol dependence in a large case/control data set [15].

Another issue concerns whether or not the present family-based findings did, indeed, replicate the top GWAS SNPs reported by Zuo et al. [15] and Wang *et al*. [12]. Of the five SNPs reported as reaching genome-wide significance, two SNPs, rs10912899 and rs2269650, in the present study were within 300 – 400 base pairs of two SNPs reported by Zuo *et al*. [15] and Wang *et al*. [12] to have genome-wide significance (rs6701037 and rs1057302). It is noteworthy that Wang *et al*. [12] reporting on their meta-analytic family study data were not able to completely replicate individual SNPs from the GWAS findings. Of the four SNPs reported by Wang *et al*. [12], one was not significant, and two had nominal p-values, though one was highly significant.

The issue of representativeness of our findings may be considered a limitation. The multiplex families on which the present report is based were ascertained through affected sib pairs. Multiplex families appear to differ from alcohol dependent families in the general population by having greater transmission of alcohol dependence across generations. Follow-up of third generation offspring from multiplex families shows an exceptionally high rate of AD and associated substance use by young adulthood [39,40]. This suggests that multiplex family samples may provide an efficient means of identifying genes because of the greater likelihood that genes may be segregating within these families that confer greater susceptibility to early onset alcohol dependence and related substance use disorders [41].

Another possible limitation of our results is that some of the diagnoses were based on DSM-III criteria and others on DSM-IV. The DSM-III system was the current system in place when Generation I and II was recruited. The definition of alcohol dependence provided by DSM-III requires the presence of tolerance and physical dependence. With the initiation of a longitudinal follow-up for Generation III, subjects were assessed using DSM-IV criteria. These criteria require three or more symptoms within a 12-month period that may include tolerance or physical dependence but do not require the presence of these symptoms if other symptoms are present. Accordingly, the third generation may have met criteria for alcohol dependence based on social or occupational impairment, use in spite of physical impairment, persistent desire to use, or inability to cut down or control use.

An additional limitation of our analysis was the need to include a broader phenotypic definition for the Generation III individuals. While it would be possible to code any Generation III individual as unaffected if they did not meet criteria for alcohol dependence, it appeared that this would incorrectly reflect individual’s addiction susceptibility where significant abuse or dependence on drugs was present. An additional analysis in which the third generation offspring was coded for alcohol dependence only (yes/no) provided essentially the same results.

Because the power to detect association increases with the number of observations available for related individuals [42], the family-based association analysis was strengthened by having a larger number of family members that includes three generations. However, the younger age (mean of 24 years) of the third generation family members means that not all have moved through the period of risk. As a result, some individuals coded as not dependent may eventually convert to affected status. However, in spite of this limitation we observed a significant relationship between SNPs within the KIAA0040 gene and alcohol dependence suggesting that this gene may have clinical importance in the etiology of alcohol dependence.

A comment is needed regarding the family-based approach which was taken in this report. Risch and Merikangas [43] were among the first to suggest that association studies are sometimes more powerful than linkage analyses. Since that time, there has been a shift toward large genome-wide association studies (GWAS) instead of family-based methods where sample sizes are more modest. Some have questioned whether GWAS methods that are designed to detect common rather than rare variants will explain a substantial portion of heritability in psychiatric disorders [44]. This view has been amplified by others who argue that GWAS may detect common variants with statistically significant results but only modest population attributable risk in comparison to focused investigations of families where genes can be found with high predictive value [45]. Perhaps, the use of multiple statistical genetic methods is preferable when characterizing the genetic underpinnings of complex phenotypes such as alcohol dependence. Simulations carried out using linkage, case-control association and family-based tests have shown that each method has limitations that may be handled best by the use of multiple methods [46].

In summary, the present results using family-based association found evidence that the KIAA0040 gene is related to risk for alcohol dependence and supports the GWAS results offered by Zuo *et al*. [15]. Future work is needed to uncover the function of this gene and its potential role in the risk/protection from alcohol dependence.

## Figures and Tables

**Table 1:** Results od FBAT and GC analyses for alcohol dependence in generations I and II with any substance use disorder coded as positive for generation III.

Marker	cM	Informativef amilies FBAT	FBAT p-value^c^	GC p-value	Major/ Minor Allele^a^	Nucleotide^b^	Major Allele Frequency	Minor Allele Frequency	Allele Freq. Affected	Allele Freq. Unaffected	Gene	Location
rs12094153	184.7521	44	0.591	0.584	1	G	G = 0.522	A = 0.478	0.603	0.397	*TNN*	
rs4651322	184.7578	41	0.438	0.310	1	C	C = 0.673	T = 0.327	0.615	0.385	*TNN*	
rs12563833	184.7603	40	0.065	0.051	2	A	G = 0.743	A =0.257	0.289	0.711	*TNN*	
rs1018829	184.7605	35	0.086	0.389	1	**G**	C = 0.832	T = 0.168	0.770	0.230	*TNN*	
rs2018318	184.7636	33	0.177	0.488	1	**C**	G = 0.826	T = 0.174	0.791	0.209	*TNN*	
rs6701037	184.7660	43	0.526	0.512	1	A	C = 0.518	A = 0.482	0.566	0.434		
**rs10912899**	184.7663	44	**0.021**	0.255	2	**T**	G = 0.650	A = 0.350	0.411	0.589		
rs6425323	184.7686	43	0.469	0.506	1	C	T = 0.518	C = 0.482	0.570	0.430		
rs1057302	184.7693	43	0.469	0.540	1	**A**	C = 0.513	T = 0.487	0.575	0.425	*KIAA*0040	*Exon* 5
**rs2269650**	184.7715	45	**0.037**	0.076	2	A	C = 0.677	A = 0.323	0.336	0.664	*KIAA*0040	***Exon* 5**
rs1057239	184.7719	43	0.604	0.160	1	**G**	T = 0.509	C = 0.491	0.559	0.441	*KIAA*0040	*Exon* 5
rs1894709	184.7735	43	0.497	0.098	1	**G**	A = 0.509	C = 0.491	0.557	0.443	*KIAA*0040	*Intron* 4
rs2269655	184.7759	35	0.272	0.617	1	**G**	G = 0.881	T = 0.119	0.824	0.818	*KIAA*0040	
**rs2861158**	184.7761	37	**0.033**	0.052	2	A	G = 0.743	A = 0.257	0.245	0.755	*KIAA*0040	***Exon* 4**
rs16847872	184.7780	35	0.266	0.648	1	**T**	A = 0.881	G = 0.119	0.836	0.164	*KIAA*0040	*Intron* 3
**rs1008459**	184.7877	37	**0.006**	**0.013**	2	G	A = .0735	G = 0.265	0.268	0.732	*KIAA*0040	***Intron* 2**
**rs2272785**	184.7955	38	**0.015**	**0.031**	2	A	C = 0.712	A = 0.288	0.268	0.733	*KIAA*0040	***Intron* 2**
**rs3753555**	184.7964	45	**0.021**	0.082	2	**G**	A = 0.588	C = 0.422	0.448	0.552		

a 1, major and 2, minor allele (designation based on NCBI build 37.3 allele frequencies for European Caucasians).

b Bolded nucleotides are the reverse strand.

c Using a more restrictive phenotype for Generation III that included only alcohol abuse and alcohol dependence p-values are: rs10912899, p = 0.035; rs2269650, p = 0.033; rs2861158, p = 0.037; rs1008459, p = 0.014; rs2272785, p = 0.062; rs3753555, p = 0.020.

**Table 2:** Diagnostic status by generation.

	Age Mean ± SD	Affected	Unaffected	Unknown	Total	Genotyped
Generation I	59.4 (9.1)	115	127	6	248	121
Alcohol Dependence						
Generation II	36.4 (7.8)	335	162	6	503	392
Alcohol Dependence						
Generation III	23.9 (5.8)	92	147	10	249	248
SUD Phenotype						
Alcohol Abuse/Dependence Phenotype		47	195	7	249	248
Total		542	436	22	1000	761

Three individuals from an ancestral generation (great grandparents of third generation subject) were also genotyped, but are not included in the tables.

**Table 3:** Gender distribution by generation for individuals with phenotype.

	Male	Female	Total
Generation I	124	124	248
Generation II	250	253	503
Generation III	115	134	249
Total	490	511	1000

**Table 4:** Haplotype analysis.

			Block		Overall	

Markers	Haplotype	Freq.	p-value^a^	p-value^b^	p-value^a^	p-value^b^
rs2272785–rs3753555	C-T	0.577	0.017	0.044	0.041	0.034
	A-G	0.238	0.006	0.009		
rs1008459–rs2272785–rs3753555	A-C-T	0.585	0.016	0.043	0.012	0.011
	G-A-G	0.205	0.002	0.003		

a Affected status for Generation III includes any SUD;

b Affected status for Generation III is alcohol dependence only.

## References

[1] Mokdad AH, Marks JS, Stroup DF, Gerberding JL (2004). Actual causes of death in the United States, 2000. JAMA.

[2] Harwood H (2000). Updating estimates of the economic costs of alcohol abuse in the United States: Estimates, update methods, and data. Report prepared by The Lewin Group for the National Institute on Alcohol Abuse and Alcoholism. Based on estimates, analyses, and data reported in Harwood H, Fountain D, Livermore G. The economic costs of alcohol and drug abuse in the United States 1992. Report prepared for the National Institute on Drug Abuse and the National Institute on Alcohol Abuse and Alcoholism, National Institutes of Health, Department of Health and Human Services.

[3] Grant BF, Dawson DA, Stinson FS, Chou SP, Dufour MC, Pickering RP (2004). The 12-month prevalence and trends in DSM-IV alcohol abuse and dependence: United States, 1991–1992 and 2001–2002. Drug Alcohol Dependence.

[4] Hasin DS, Stinson FS, Ogburn E, Grant BF (2007). Prevalence, correlates, disability, and comorbidity of DSM-IV alcohol abuse and dependence in the United States: results from the National Epidemiologic Survey on alcohol and related conditions. Archives of General Psychiatry.

[5] Kessler RC, Crum RM, Warner LA, Nelson CB, Schulenberg J, Anthony J (1997). Lifetime co-occurrence of DSM-III-R alcohol abuse and dependence with other psychiatric disorder in the national comorbidity survey. Archives of General Psychiatry.

[6] Caldwell CB, Gottesman II (1991). Sex differences in the risk for alcoholism: A twin study. Behavior Genetics.

[7] Heath AC, Bucholz KK, Madden PAF, Dinwiddie SH, Slutske WS (1997). Genetic and environmental contributions to alcohol dependence risk in a national twin sample: Consistency of findings in women and men. Psychological Medicine.

[8] Prescott CA, Aggen SH, Kendler KS (1999). Sex differences in the sources of genetic liability to alcohol abuse and dependence in a population based sample of US twins. Alcoholism: Clinical and Experimental Research.

[9] Kendler KS, Heath AC, Neale MC, Kessler RC, Eaves LJ (1992). A population-based twin study of alcoholism in women. JAMA.

[10] Reich T, Edenberg HJ, Goate A, Williams JT, Rice JP (1998). Genome-wide search for genes affecting the risk for alcohol dependence. American Journal of Medical Genetics Part B: Neuropsychiatr Genet.

[11] Edenberg HJ, Dick DM, Xuei X, Tian H, Almasy L (2004). Variations in GABRA2, encoding the alpha 2 subunit of the GABA(A) receptor, are associated with alcohol dependence and with brain oscillations. The American Journal of Human Genetics.

[12] Wang KS, Liu X, Zhang Q, Pan Y, Aragam N, Zeng M (2011). A meta-analysis of two genome-wide association studies identifies 3 new loci for alcohol dependence. Journal of Psychiatric Research.

[13] Hill SY, Shen S, Zezza N, Hoffman EK, Perlin M, Allan W (2004). A genome-wide search for alcoholism susceptibility genes. Am J Med Genet, Part B.

[14] Hill SY, Weeks DE, Jones BL, Zezza N, Stiffler S (2012). ASTN1 and Alcohol Dependence: Family-Based Association Analysis in Multiplex Alcohol Dependence Families. American Journal of Medical Genetics Part B.

[15] Zuo L, Gelernter J, Zhang CK, Zhao H, Lu L (2012). Genome-Wide association study of alcohol dependence implicates *KIAA0040* on Chromosome 1q. Neuropsychopharmacology.

[16] Helzer JE, Robins LN, McEvoy LT, Spitznagel EL, Stoltzman RK (1985). A comparison of clinical and diagnostic interview schedule diagnoses. Physician reexamination of lay-interviewed cases in the general population. Archives of General Psychiatry.

[17] American Psychiatric Association (1980). Diagnostic and Statistical Manual of Mental Disorders (DSM-III).

[18] American Psychiatric Association (1987). Diagnostic and Statistical Manual of Mental Disorders-Revised (DSM-III-R).

[19] Feighner JP, Robins E, Guze SB, Woodruff RA, Winokur G, Munoz R (1972). Diagnostic criteria for use in psychiatric research. Archives of General Psychiatry.

[20] Janca A, Robins LN, Cottler LB, Early TS (1992). Clinical observation of assessment using the Composite International Diagnostic Interview (CIDI). An analysis of the CIDI field trials B wave II at the St. Louis site. The British Journal of Psychiatry.

[21] Cottler LB, Robins LN, Helzer JE (1989). The reliability of the CIDI-SAM: A comprehensive substance abuse interview. British Journal of Addiction.

[22] Tellegen A, Lykken DT, Bouchard TJ, Wilcox KJ, Segal NL, Rich S (1988). Personality similarity in twins reared apart and together. Journal of Personality and Social Psychology.

[23] O’Connell JR, Weeks DE (1998). PedCheck: A program for identifying genotype incompatibilities in linkage analysis. The American Journal of Human Genetics.

[24] Lange K, Cantor R, Horvath S, Perola M, Sabatti C (2001). Mendel version 4.0: A complete package for the exact genetic analysis of discrete traits in pedigree and population data sets. The American Journal of Human Genetics.

[25] Mukhopadhyay N, Almasy L, Schroeder M, Mulvihill WP, Weeks DE (2005). Mega2: data-handling for facilitating genetic linkage and association analyses. Bioinformatics.

[26] Mukhopadhyay N, Tang X, Weeks DE (2010). Genetic Map Interpolator. Annual Meeting of the American Society of Human Genetics.

[27] Kong X, Murphy K, Raj T, He C, White PS, Matise TC (2004). A combined linkage-physical map of the human genome. The American Journal of Human Genetics.

[28] Matise TC, Chen F, Chen W, De La Vega FM, Hansen M (2007). A second-generation combined linkage physical map of the human genome. Genome Research.

[29] Laird NM, Horvath S, Xu X (2000). Implementing a unified approach to family-based tests of association. Genetic Epidemiology.

[30] Rabinowitz D, Laird N (2000). A unified approach to adjusting association tests for population admixture with arbitrary pedigree structure and arbitrary missing marker information. Human Heredity.

[31] Spielman RS, McGinnis RE, Ewens WJ (1993). Transmission test for linkage disequilibrium: The insulin gene region and insulin-dependent diabetes mellitus (IDDM). The American Journal of Human Genetics.

[32] Sinsheimer JS, Blangero J, Lange K (2000). Gamete-competition models. The American Journal of Human Genetics.

[33] Barrett JC, Fry B, Maller J, Daly MJ (2005). HAPLOVIEW: Analysis and visualization of LD and haplo-type maps. Bioinformatics.

[34] Gabriel SB, Schaffner SF, Nguyen H, Moore JM, Roy J (2002). The structure of haplotype blocks in the human genome. Science.

[35] Horvath S, Xu X, Lake SL, Silverman EK, Weiss ST, Laird NM (2004). Family-based tests for associating haplotypes with general phenotype data: Application to asthma genetics. Genetic Epidemiology.

[36] Pruim RJ, Welch RP, Sanna S, Teslovich TM, Chines PS (2010). LocusZoom: Regional visualization of genome-wide association scan results. Bioinformatics.

[37] Peng W, Wang HY, Miyahara Y, Peng G, Wang R-F (2008). Tumor-associated galectin-3 modulats the function of tumor-reactive T cells. Cancer Research.

[38] Neidhardt J, Fehr S, Kutsche M, Lohler J, Schachner M (2003). Tenascin-N: Characterization of a novel member of the tenascin family that mediates neurite repulsion from hippocampal explants. Molecular and Cellular Neuroscience.

[39] Hill SY, Shen S, Locke-Wellman J, Matthews AG, McDermott M (2008). Psychopathology in offspring from multiplex alcohol dependence families: A prospective study during childhood and adolescence. Psychiatry Research.

[40] Hill SY, Tessner KD, McDermott MD (2011). Psychopathology in offspring from families of alcohol dependent female probands: A prospective study. Journal of Psychiatric Research.

[41] Hill SY, Reilly MT, Lovinger DM (2010). Neural Plasticity, human genetics, and risk for alcohol dependence. Functional Plasticity and Genetic Variation.

[42] Sahana G, Guldbrandtsen B, Janss C, Ott J (2010). Comparison of association mapping methods in a complex pedigree population. Genetic Epidemiology.

[43] Risch N, Merikangas K (1996). The future of genetic studies of complex human diseases. Science.

[44] Maher B (2008). Personal genomes: The case of missing heritability. Nature.

[45] Mitchell KJ, Porteous DJ (2009). GWAS for psychiatric disease: Is the framework built on a solid foundation. Molecular Psychiatry.

[46] Suarez BK, Culverhouse R, Jin CH, Hinrichs A (2007). Linkage, case-control association, and family-based association tests for complex disorders. BMS Proceedings.

